# In Situ Identification of Spin Magnetic Effect on Oxygen Evolution Reaction Unveiled by X-ray Emission Spectroscopy

**DOI:** 10.1021/jacs.4c18149

**Published:** 2025-04-12

**Authors:** Chih-Ying Huang, Hsin-An Chen, Wei-Xuan Lin, Kuan-Hung Chen, Yu-Chang Lin, Tai-Sing Wu, Chia-Che Chang, Chih-Wen Pao, Wei-Tsung Chuang, Jyh-Chyuan Jan, Yu-Cheng Shao, Nozomu Hiraoka, Jau-Wern Chiou, Pai-Chia Kuo, Jessie Shiue, Deepak Vishnu S. K, Raman Sankar, Zih-Wei Cyue, Way-Faung Pong, Chun-Wei Chen

**Affiliations:** † International Graduate Program of Molecular Science and Technology (NTU-MST), National Taiwan University, Taipei 106319, Taiwan; ‡ Molecular Science and Technology Program Taiwan International Graduate Program (TIGP), Academia Sinica, Taipei 115201, Taiwan; § Institute of Materials Science and Engineering, National Taipei University of Technology, Taipei 10608, Taiwan; ∥ Department of Physics, Tamkang University, New Taipei City 251301, Taiwan; ⊥ National Synchrotron Radiation Research Center, Hsinchu 300, Taiwan; # Department of Applied Physics, National University of Kaohsiung, Kaohsiung 811, Taiwan; ¶ Institute of Atomic and Molecular Sciences, Academia Sinica, Taipei 106319, Taiwan; ∇ Department of Chemistry, National Tsing Hua University, Hsinchu 300, Taiwan; ○ Institute of Physics, Academia Sinica, Taipei 115201, Taiwan; ⧫ Department of Materials Science and Engineering, National Taiwan University, Taipei 106319, Taiwan; †† Center for Condensed Matter Sciences and Center of Atomic Initiative for New Materials (AI-MAT), National Taiwan University (NTU), Taipei 106319, Taiwan

## Abstract

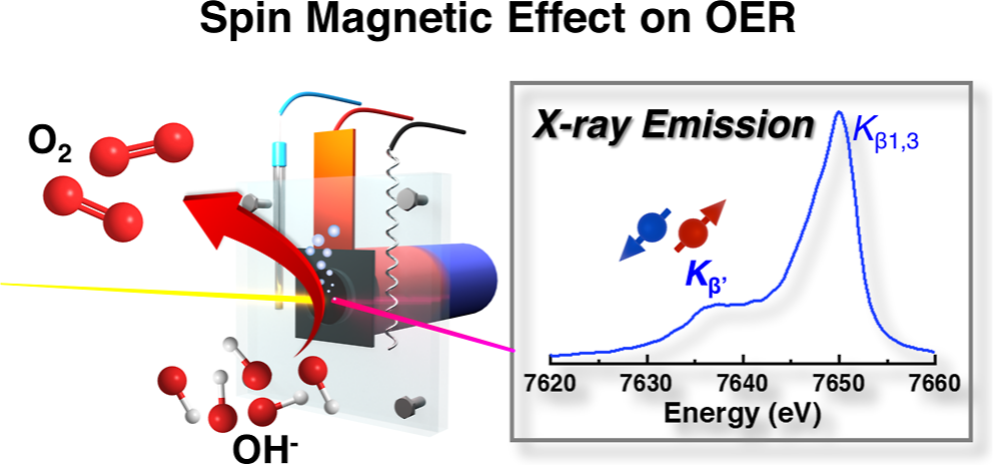

Manipulating the spin ordering of the oxygen evolution reaction (OER) catalysts through magnetization has recently emerged as a promising strategy to enhance performance. Despite numerous experiments elaborating on the spin magnetic effect for improved OER, the origin of this phenomenon remains largely unexplored, primarily due to the difficulty in directly distinguishing the spin states of electrocatalysts during chemical reactions at the atomic level. X-ray emission spectroscopy (XES), which provides information sensitive to the spin states of specific elements in a complex, may serve as a promising technique to differentiate the onset of OER catalytic activities from the influence of spin states. In this work, we employ the in situ XES technique, along with X-ray absorption spectroscopy (XAS), to investigate the interplay between atomic/electronic structures, spin states, and OER catalytic activities of the CoFe_2_O_4_ (CFO) catalyst under an external magnetic field. This enhancement is due to the spin magnetic effect that facilitates spin-selective electron transfer from adsorbed OH^–^ reactants, which strongly depends on the spin configurations of the tetrahedral-(*T*_*d*_) and octahedral-(*O*_*h*_) sites of both Fe and Co ions. Our result contributes to a comprehensive understanding of magnetic field-assisted electrocatalysis at the atomic level and paves the way for designing highly efficient OER catalysts.

## 1 Introduction

Electrochemical water splitting, comprising hydrogen evolution reaction (HER) and oxygen evolution reaction (OER), is a highly promising technology for generating clean hydrogen essential for sustainable energy conversion.^1,2^ The major challenge lies in finding efficient, cost-effective, and durable catalysts to enhance the cathodic HER and anodic OER at low overpotentials. In particular, the high activation barriers and sluggish kinetics of OER limit the overall efficiency of water electrolysis, so there is an urgent demand for low-cost, efficient, and stable catalysts for OER.^3,4^ The general strategy to design electrocatalysts with improved performances is either increasing the number of active sites^5^ or enhancing the intrinsic activity of individual active sites.^6^ Because the ground state of dioxygen is a spin triplet with about 1 eV lower than the excited state of dioxygen and the reactants (H_2_O and OH^–^),^7^ which are a spin singlet, manipulation of electronic spin has recently emerged as a promising strategy to boost the performance of OER catalysts.^8^ Spin-polarized electrons on the catalyst surface play a crucial role in promoting the generation of parallel spin-aligned oxygens, thereby enhancing the performance of the OER. This enhancement is often observed when interatomic ferromagnetic (FM) interactions of the catalyst dominate, which facilitates the spin-selective electron transfer from singlet reactants to form triplet dioxygen. Consequently, this mechanism leads to improved catalytic activity, as explained by the theory of quantum spin-exchange interactions (QSEI).^9^ Further magnetization by an external magnetic field may increase the spin polarization of FM catalysts, thus enhancing the OER performance.

Several research groups have experimentally demonstrated the spin magnetic effect on OER. They have shown that applying a direct magnetic field can enhance electrocatalytic water oxidation, with the enhancement positively correlated with magnetization.^10−13^ For example, Galán-Mascarós et al. reported that the OER activity of group VIII metal oxides, such as NiZnFeO_*x*_, is significantly enhanced when an external field is applied.^14^ Xu et al. revealed that spin polarization induced by a magnetic field on FM CoFe_2_O_4_ (CFO) may facilitate the formation of triplet dioxygen in the OER. By contrast, this phenomenon does not apply to non-FM catalysts such as antiferromagnetic (AFM) Co_3_O_4_ or paramagnetic IrO_2_.^15^ They also reported that the disappearance of domain walls in an FM material may enhance spin-polarized OER, suggesting that single-domain FM particles can achieve maximum performance.^16^ Zhao et al. demonstrated a significant increase in the OER current of ferrimagnetic Fe_3_O_4_ under an external magnetic field. They found that the synergistic spin-enhanced singlet O–H cleavage and triplet O–O bonding during molecular water oxidation (in a weak alkaline solution) results in a more pronounced OER acceleration than the oxidation of OH^–^ with only O–O bonding (in a strong alkaline solution).^17^ Xu et al. studied the role of coercivity in electrocatalytic OER with single-domain CFO nanocrystals in the presence of a magnetic field. Their findings suggest that a CFO with higher coercivity and a more ordered spin polarization state promotes more efficient electron transfer. Additionally, the magnetic field-assisted OER activity is further enhanced as the coercivity increases.^16^

Understanding and harnessing the spin magnetic effect in the OER catalysis represent promising avenues for developing next-generation electrocatalysts with promising activity and stability for sustainable energy conversion applications. Despite numerous reported experiments elaborating on the spin magnetic effect for enhanced OER, the origin of this phenomenon is far from being fully understood. This complexity is mainly attributed to the difficulty in directly distinguishing the spin states of electrocatalysts during the chemical reaction at the atomic level.^18,19^ Recently, the in situ/operando identification of atomic or electronic configurations of catalysts at the solid–liquid interface has emerged as a valuable strategy to address the complexities of electrocatalytic reactions.^20^ In particular, in situ X-ray absorption spectroscopy (XAS) has become one of the most popular techniques for obtaining valuable insights into the atomic-scale understanding of electrocatalyst configurations and structures during chemical reactions. Specifically, X-ray absorption near-edge structure (XANES) spectra can provide information about the electronic structures of specific elements, while extended X-ray absorption fine structure (EXAFS) spectra may offer detailed information regarding the local coordination environment.^21,22^

Despite many successful experimental reports using in situ XAS to understand electrocatalytic chemical reactions,^21,23^ it remains challenging to use these methods to ascertain the extent to which electrocatalytic activities are influenced by spin modification. By contrast, hard X-ray emission spectroscopy (XES), which provides information sensitive to the spin states of specific elements in a complex, may serve as a promising technique to differentiate OER catalytic activities from the influence of spin states.^24−28^ In this work, we aim to employ the in situ XES technique, along with XAS, to probe the relationships among atomic/electronic structures, spin states, and the OER catalytic activities of the CFO catalyst under an external magnetic field. The CFO has been widely studied as a model system to illustrate the magnetic enhancement of catalytic activities in the OER.^15,16,29,30^ The correlations between the electronic structures and the spin states of Fe and Co ions revealed by in situ XAS and XES shed light on the processes of spin-specific electron transfer and the spin magnetic effect. Our results offer direct evidence in delineating the interplay between atomic/electronic structures and spin configurations of both Fe and Co ions within the CFO for enhanced OER efficiencies when subjected to an external magnetic field. This contributes to a comprehensive insight into magnetic field-assisted electrocatalysis and facilitates the design and synthesis of highly effective catalysts for the OER.

## 2 Results and Discussion

FM materials exhibit a parallel alignment of magnetic moments within their atomic domains, resulting in a strong net magnetic moment. Conversely, ferrimagnetic materials have magnetic moments aligned antiparallel but with unequal magnitudes, leading to a net magnetic moment that is typically weaker than that of FM materials.^31^ CFO, which has an inverse-spinel crystal structure, is ferrimagnetic, similar to Fe_3_O_4_.^32,33^ In CFO, Fe and Co ions typically occupy the tetrahedral (*T*_*d*_) and octahedral (*O*_*h*_) sublattices, aligning antiparallel to each other to create a ferrimagnetic order with nonzero magnetization at room temperature. Here, CFO nanocrystals were synthesized via a hydrothermal method, as described in the experimental section.^16^

**Figure 1:**
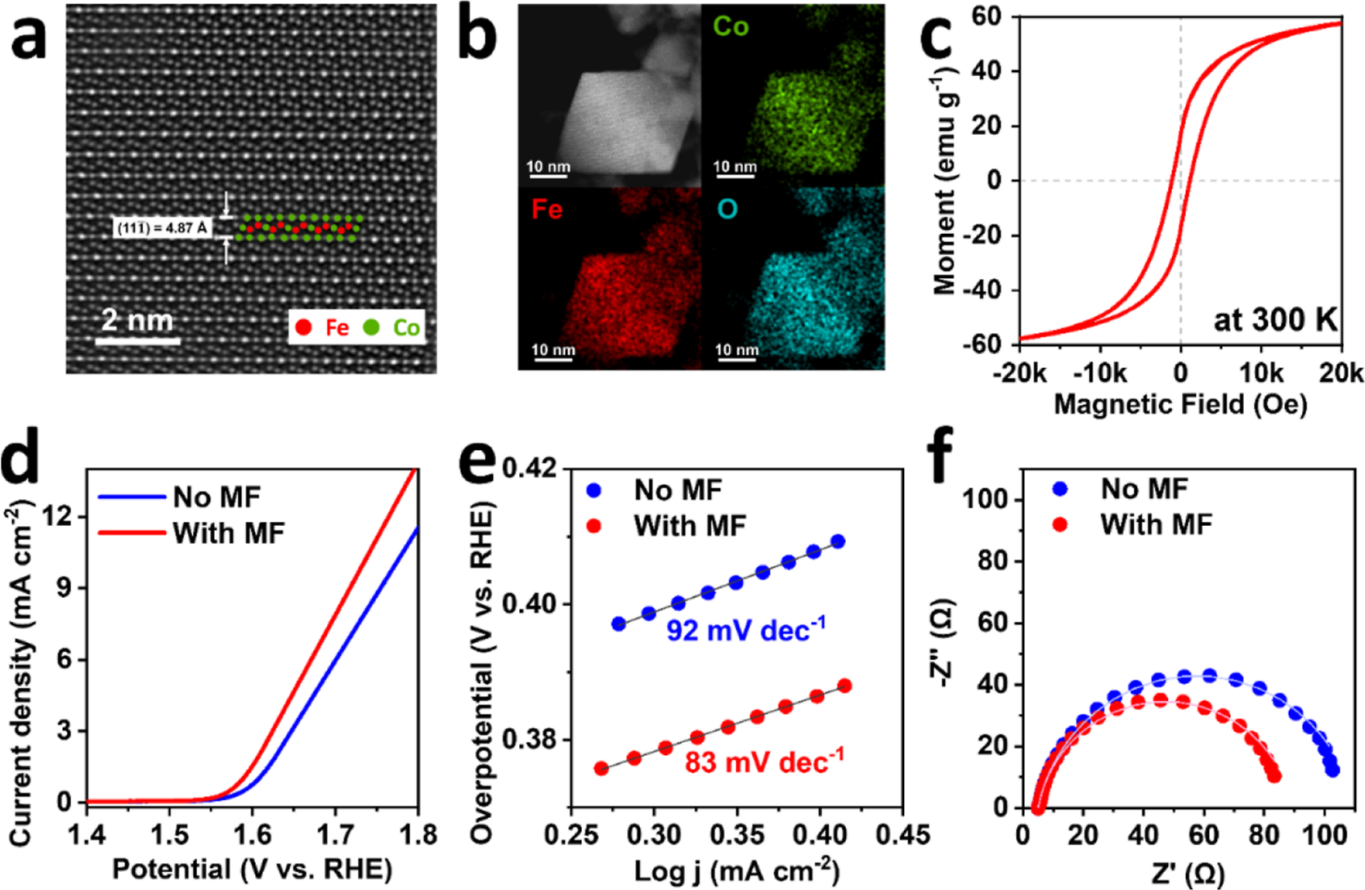
(a) STEM and (b) STEM energy-dispersive X-ray spectroscopy (STEM-EDS) mapping images CFO. (c) Hysteresis loops of CFO measured at 300 K. (d) LSV curves, (e) Tafel slopes, and (f) Nyquist plots of CFO recorded in 1 M KOH electrolyte with and without magnetic field applied.

The aberration-corrected scanning transmission electron microscopy (STEM) provides atomic imaging and confirms the well-crystalline feature of CFO. Figure 1a shows the atomic resolution STEM image of the CFO along the (110) axis, where the bright dots are Co and Fe atoms. The measured d-spacing of 4.87 Å corresponds to the (111̅) plane of the face-centered cubic CFO structure.^34^ In addition, X-ray diffraction (XRD) analysis was conducted to verify the crystal structures, and the obtained diffraction pattern corresponds to the cubic CFO inverse spinel (PDF #04-006-4148), as shown in Figure S1.^16,34^ The structure of CFO is further affirmed by Raman spectra, with the peak positioned at 475 cm^–1^, associated with the vibration of oxygen near the *O*_*h*_ site and the peak positioned at 684 cm^–1^, attributed to the vibration of oxygen near the *T*_*d*_ site as shown in Figure S1.^16^

Figure 1b exhibits the elemental distribution of CFO obtained by STEM energy-dispersive X-ray spectroscopy (STEM-EDS) analysis. The STEM-EDS mapping images indicate that there is a homogeneous distribution of all the elements, including Fe, Co, and O in CFO nanocrystals, and the corresponding Co/Fe/O ratio of CFO nanocrystals is approximately 11%:26%:63% as revealed by the EDS analysis (Figure S2). Figure 1c illustrates the M–H hysteresis loop of CFO measured at 300 K, confirming its room-temperature ferrimagnetic behavior with a saturation magnetization of approximately 57 emu g^–1^, which is similar to the previous report.^15^ The primary object of this study is to use in situ XAS and XES techniques to investigate the spin magnetic effect of the CFO catalyst during the OER in the presence of a magnetic field. To accomplish this, we need to integrate a magnet into the limited space of the in situ measurement setup, as illustrated in the following section. Hence, we employed a customized electrochemical cell equipped with a permanent magnet to assess the enhanced efficiency of the OER in a 1 M KOH electrolyte under a magnetic field strength of up to 0.4 T. This approach may differ from previous reports that employed an electromagnet to conduct the magnetic enhancement OER experiment.^15,16^Figure 1d exhibits the OER activities of the CFO catalyst with and without the application of a magnetic field. The OER performance of the CFO catalyst under a magnetic field of 0.4 T demonstrates a reduction in overpotential by 39 mV at 10 mA cm^–2^ compared with that without a magnetic field. Figure 1e shows the corresponding Tafel slopes of CFO catalysts, which are 83 and 92 mV dec^–1^ for the CFO catalysts with and without a magnetic field, respectively. Furthermore, the Nyquist plots obtained from electrochemical impedance spectroscopy (EIS) tests reveal that applying a magnetic field may reduce the charge transfer resistance (*R*_ct_). Figure 1f shows that the CFO with a magnetic field possesses a smaller charge transfer resistance *R*_ct_ (84 Ω) than the CFO without a magnetic field (105 Ω), indicating that applying a magnetic field may cause enhanced OER catalytic activities of the CFO. Our result on the magnetically enhanced OER with the CFO catalyst is comparable to those obtained in the previous reports,^15,35^ performed under similar experimental conditions. In addition, the details of EIS are provided in the Supporting Information, Tables S1 and S2.

**Figure 2:**
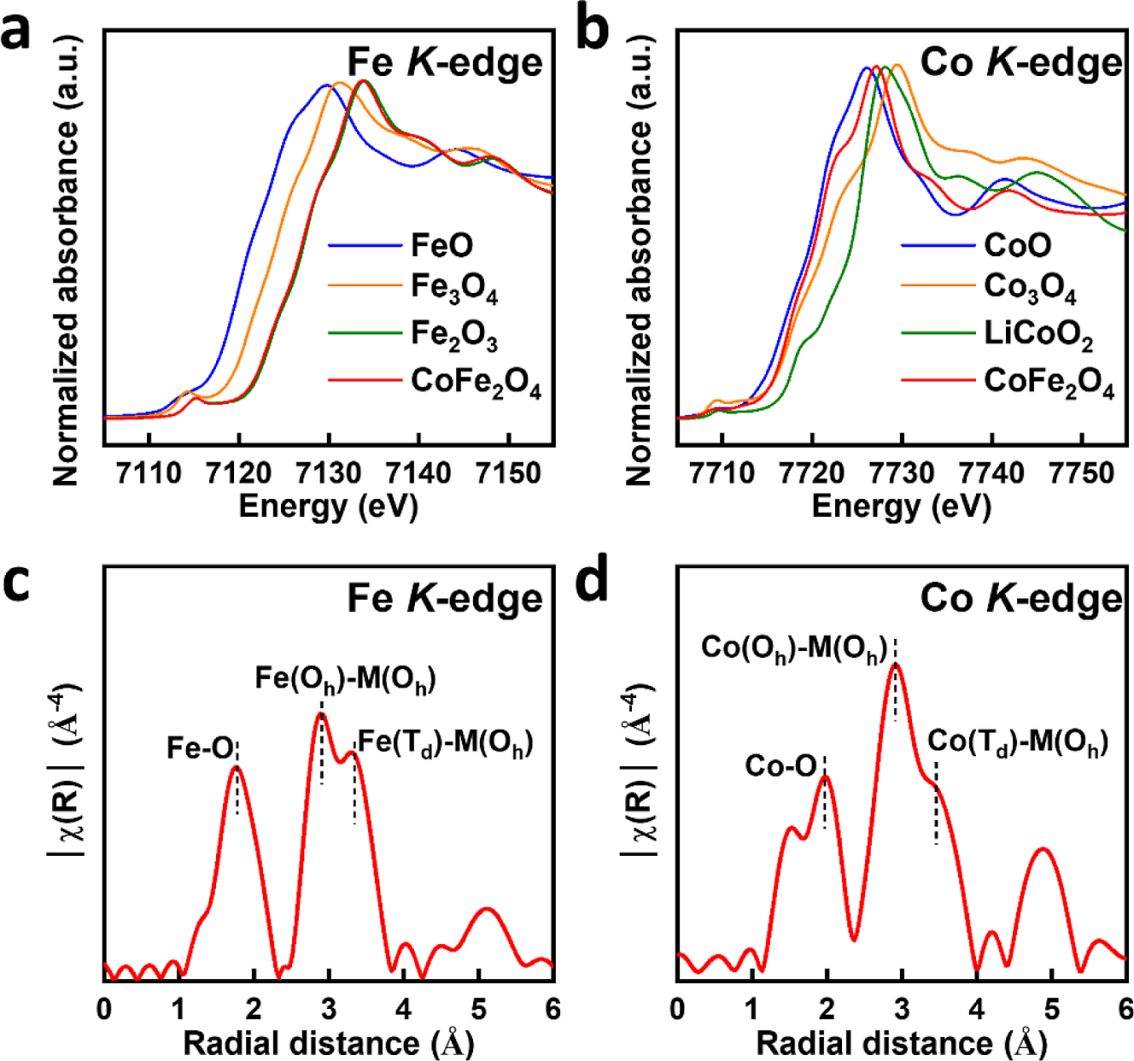
(a) Normalized Fe and (b) Co K-edge XANES spectra of CFO compared with references. Phase-corrected FT-EXAFS spectra of (c) Fe and (d) Co K-edge of CFO obtained from *k*^3^-weighted.

Next, we perform XAS analyses to investigate the atomic and electronic structures of the Fe and Co sites of the CFO catalyst. All XAS analyses were operated at the Taiwan Photon Source (TPS) 44A beamline at the National Synchrotron Radiation Research Center (NSRRC), Taiwan (experimental details can be found in the Supporting Information). Figure 2a,b presents the Fe (Co) K-edge XANES spectra of CFO and the reference samples corresponding to FeO, Fe_3_O_4_, and Fe_2_O_3_ (CoO, Co_3_O_4_, and LiCoO_2_). According to the dipole-transition selection law, these Fe (Co) K-edge XANES spectra are primarily associated with the Fe (Co) 1s → 4p transition, and the intensity of the main absorption feature is attributed to the density of the unoccupied Fe (Co) 4p states. All the spectra in Figure 2a,b have a common weak pre-edge feature at the Fe (Co) K-edge, whereas the additional weak feature is governed by the Fe (Co) 1s → 3d quadrupole transition.^36−38^ As displayed in Figure 2a, the energy threshold of the Fe K-edge feature of pristine CFO is at ∼7126.0 eV, which is above those of FeO (Fe^2+^), Fe_3_O_4_ (Fe^8/3+^), and is close to that of Fe_2_O_3_ (Fe^3+^).^39^ By contrast, the energy threshold of the Co K-edge absorption feature of CFO is located at ∼7120.5 eV, which is below those of Co_3_O_4_ (Co^8/3+^), LiCoO_2_ (Co^3+^) and is close to that of CoO (Co^2+^),^40^ as shown in Figure 2b. The valence states of Fe (Co) in CFO were further quantitatively determined through the interpolation method by the calibration curve obtained linearly from the reference samples, as shown in the Supporting Information. The valence state of Fe ions in CFO is estimated to be approximately 2.9, whereas the valence state of Co ions is around 2.1, according to the calibration curves of Fe and Co in CFO plotted in Figure S3.

CFO has an inverse-spinel crystal structure similar to that of Fe_3_O_4_ and is typically represented by the chemical formula (Fe^3+^)_A_[Fe^2+^Fe^3+^]_B_O_4_, where the parentheses and square brackets refer to tetrahedral-A (*T*_*d*_) and octahedral-B (*O*_*h*_) sites, respectively.^41^Figure 2c,d shows the Fourier transform (FT) spectra of Fe/Co K-edge EXAFS spectra of CFO to elucidate the local atomic structures around the Co and Fe sites. The Fe/Co K-edge EXAFS spectra reveal main FT features (FT-EXAFS) at ∼1.9, 2.9, and 3.3 Å, which correspond to the nearest-neighbor Fe–O/Co–O, next-neighbor-neighbor Fe_oct_–M_oct_/Co_oct_–M_oct_ and Fe_tet_–M_oct_/Co_tet_–M_oct_ bond distances in the CFO. The symbol “M” refers to the cations (either Fe or Co), and the subscripts “tet” and “oct” indicate the *T*_*d*_ A- and *O*_*h*_ B-sites, respectively. Based on the intensities of the spectral features, there are more Fe cations at the *O*_*h*_-sites than that at the *T*_*d*_-sites. Fe ions occupy the *T*_*d*_ A-sites as Fe^3+^, while the remaining Fe ions are located at the *O*_*h*_ B-sites as mixed-valence ions of Fe^2+^ and Fe^3+^.^42^ Meanwhile, Co ions, which have a *d*^7^ electron configuration, are also found in both the *T*_*d*_ A- and the *O*_*h*_ B-sites due to their mixed-spinel structure. Nevertheless, Co ions are predominantly located in the *O*_*h*_ B-sites of CFO, exhibiting significantly greater intensity compared to the *T*_*d*_ A-sites.^15,33,42^

Regardless of the degree of mixed-spinel structure, the metal centers Fe and Co are typically in the high-spin state with the *T*_*d*_ and *O*_*h*_ sublattices aligned antiparallel to each other to create ferrimagnetic order with a nonvanishing magnetization in CFO.^43^ The existence of nonvanishing magnetization both for Fe and Co in CFO at room temperature will be responsible for the spin magnetic effect on enhancing the OER catalytic activities of CFO under a magnetic field, as unveiled in the following in situ XES analyses. Before performing the in situ XES analyses, we also measured the in situ XAS to examine the local atomic structures of Fe and Co ions of CFO during OER. The FT-EXAFS of Fe (Co) K-edge, as illustrated in Figure S4, unveils the similar local atomic environments of Fe and Co ions of CFO catalyst at the *T*_*d*_ A- and *O*_*h*_ B-sites under an applied bias during the OER process. There is no significant structural change in the CFO catalyst when a potential bias is applied. We also performed Raman and STEM analyses, as shown in Figures S5 and S6. Both the Raman spectra and STEM images of the CFO catalyst display no significant structural changes before and after the OER processes, consistent with previous studies.^15,16^ However, although our analyses indicate no permanent structural modifications before or after the OER process, the possibility of surface reconstruction during the OER operation cannot be entirely ruled out. This is due to the fact that the spectroscopic results from our experiment, including XAS and Raman analyses, may reflect averaged information on actual surface and bulk states. Consequently, employing more surface-sensitive analytical techniques is necessary to determine whether surface reconstruction occurs during the OER process.

Hard XES is operated by initially exciting a core electron through the absorption of an incoming X-ray, creating a core vacancy. Consequently, a valence electron undergoes a transition to occupy the core vacancy, releasing X-ray emission during the process. Hard XES is adept at revealing details about the local density of occupied states, specifically within the valence band. The emission feature *K*_β_, originating from the transition 3p to 1s, gives valuable details such as spin, oxidation state, and sensitivity to ligands.^23,44^ Typically, the *K*_β_ feature manifests as two distinct components: a strong *K*_β1,3_ main feature at higher energy and a satellite shoulder of *K*_β′_ at lower emitted energy. The satellite *K*_β′_ emission originates from the 3p–3d exchange interactions and indicates an average spin state with intensity typically correlated to the number of unpaired electrons in the transition metal.^44,45^ A higher spin contribution leads typically to a more pronounced *K*_β′_ feature, resulting in a strong correlation between the intensity of *K*_β′_ emission from the measured material and its spin state.^44,46^ Here, we employ the in situ XES technique to investigate the correlation between the OER activities and CFO catalysts’ spin states under the influence of a magnetic field.

**Figure 3:**
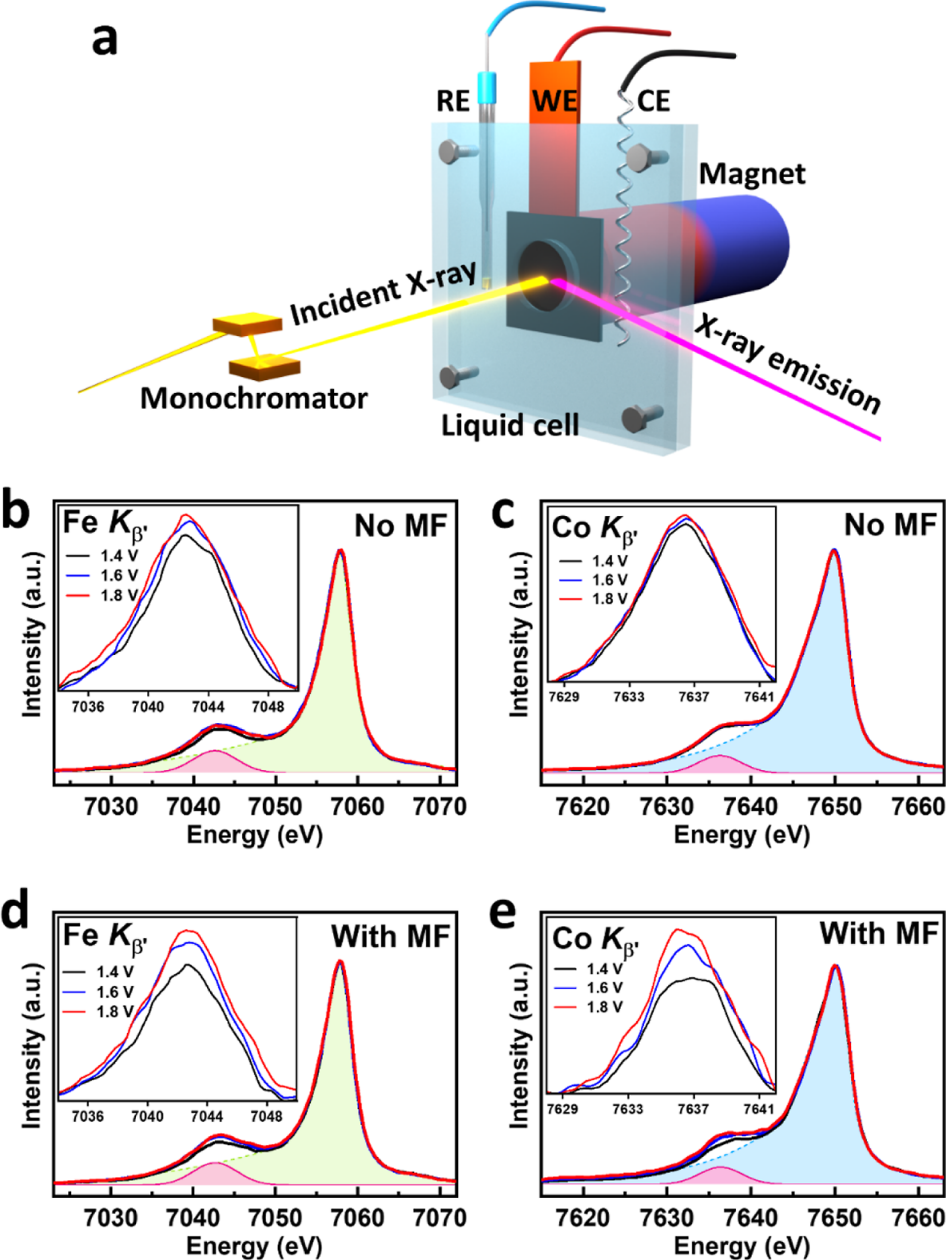
(a) Schematic of in situ electrocatalysis with a liquid cell and X-ray emission setup. The reference, working, and counter electrodes were abbreviated to RE, WE, and CE, respectively. Ag/AgCl is used as the RE, and a platinum wire as the CE. (b) Fe and (c) Co *K*_β_ XES of CFO under different potentials applied (no magnetic field, No MF), along with its fitting line as the *K*_β1,3_ by a Gaussian feature (shadow) for extracting *K*_β′_ feature (pink). (d) Fe and (e) Co *K*_β_ XES under different potentials with an applied magnetic field (With MF). The inset shows the zoom-in on the *K*_β′_ features.

Figure 3a exhibits the experimental setup of an in situ XES measurement of the CFO catalyst during the OER under a magnetic field. The Fe (Co) *K*_β_ XES of the CFO sample during the OER with and without an external magnetic field (0.4 T) was carried out at beamline BL12XU at SPring-8, Japan. Experimental details can also be found in the Supporting Information. Figure 3b,c shows the *K*_β_ emission spectra of Fe (Co) measured at 1.4 V (before onset), 1.6 V (at onset), and 1.8 V (after onset) without a magnetic field. By contrast, Figure 3d,e shows the corresponding *K*_β_ emission spectra of Fe (Co) measured at 1.4, 1.6, and 1.8 V under the influence of a magnetic field of 0.4 T. The insets of Figure 3b–e further highlight the evolution of *K*_β′_ emission spectra at different potentials of 1.4, 1.6, and 1.8 V during the OER process without and with a magnetic field. Figure S7 compares the *K*_β′_ emission spectra of Fe (Co) on the same scales. The corresponding spectra measured at reverse electrical potentials at r1.6 V and r1.4 V are also shown in Figure S8.

All these *K*_β′_ emission spectra were obtained after background subtraction using the best-fitting Gaussian curve for the feature *K*_β1,3_ as shown in the shadow. It is found that the intensities of the satellite *K*_β′_ emission features of Fe (Co) ions of CFO catalyst vary not only with an applied potential but also with an external magnetic field. As mentioned above, the satellite feature *K*_β′_ intensity is associated with the number of unpaired 3d electrons in the transition metal. Therefore, it becomes a distinct indicator of the average spin magnetic moment or spin states of Fe (Co) 3d ions. The variation in the intensities of the *K*_β′_ emission of Fe (Co) of CFO during OER in the in situ XES spectra [integrated range from 7034 to 7049 eV (7629 to 7641 eV), as shown in the insets of Figures. 3b–e] suggests that both an applied potential and an external magnetic field may cause the change of spin states of the CFO catalyst during the OER process. In addition, it is noted that the XES spectra of pristine samples and those at open-circuit potential display nearly identical profiles and *K*_β′_ intensities, as shown in Figure S9. This suggests that under the condition of no current flow, the material remains in its original state, regardless of an applied magnetic field with 0.4 T.

**Figure 4:**
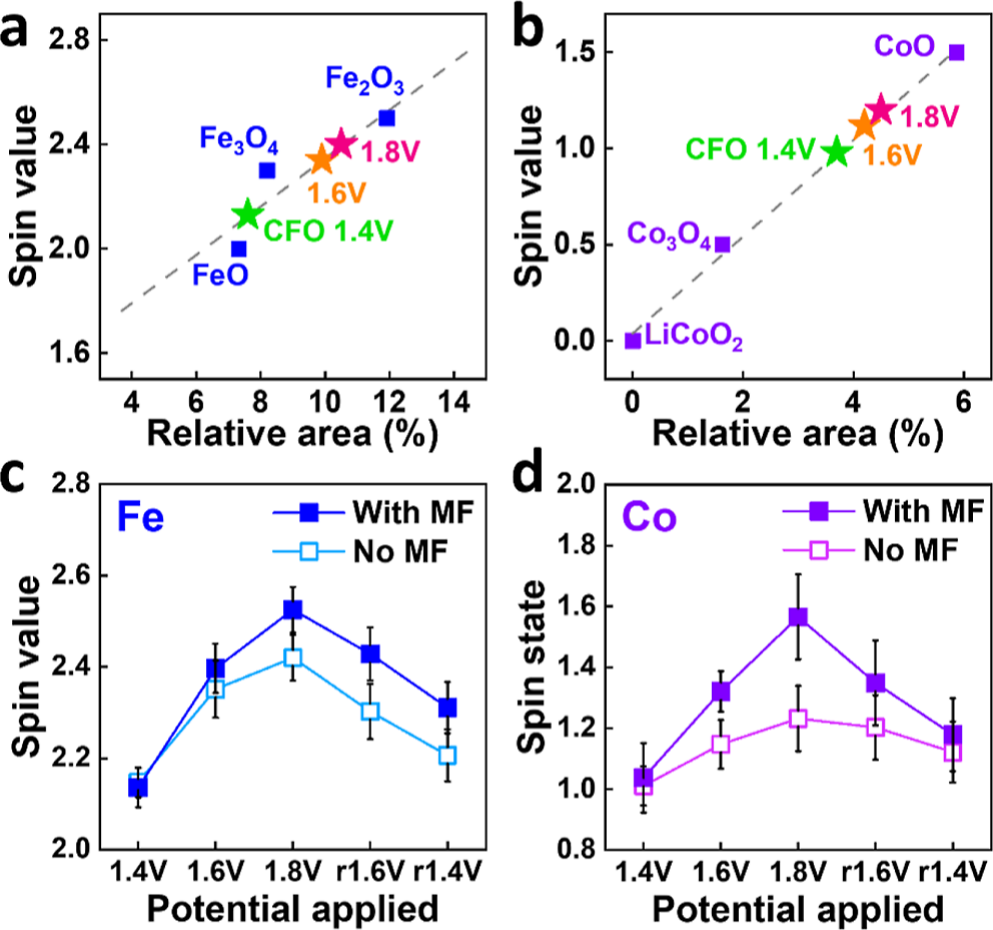
Relation between the integrated relative area of the (a) Fe and (b) Co *K*_β′_ feature regarding the overall *K*_β_, the spin state of the reference compounds, and the corresponding linear fit. (c) Integrated relative area of Fe and Co at different corresponding potentials and (d) spin states of CFO converted by interpolation of the spectral *K*_β′_ relative area in the regression line in (a,b).

To further quantify insights on the variations of spin configurations of Fe (Co) 3d ions of CFO during the OER process, a correlation correspondingly between the relative areas of Fe (Co) *K*_β′_ spectra and spin values of Fe (Co) 3d ions in CFO is established.^47^Figure 4a,b exhibits the linear calibration curves about the relationships of the proportions of the Fe (Co) XES-*K*_β′_ spectra areas to the total *K*_β_ areas with the spin values of Fe (Co) 3d ions obtained from the reference compounds of FeO, Fe_3_O_4_, and Fe_2_O_3_ (CoO, Co_3_O_4_, and LiCoO_2_).^48−51^Figure S10 shows the Fe (Co) XES-*K*_β_ spectra of all of the corresponding reference compounds. These curves were subsequently employed to estimate the spin values for both Fe and Co in CFO at different bias potentials during OER without and with an external magnetic field. The details of calculating the spin values from the obtained XES spectra with and without an external magnetic field are further described in Supporting Information. Figure 4c,d displays the evolution of the average spin values of Fe and Co 3d ions in CFO catalysts during the OER activities as a function of an applied potential bias with and without a magnetic field. The average spin values of Fe (Co) 3d ions of the CFO catalyst, which are derived from the corresponding XES-*K_β′_* intensity and the calibration curves by reference compounds,^44,47,48^ increase with increased electrical potential from 1.4 to 1.6 to 1.8 V. By contrast, the change of the spin values of Fe (Co) 3d ions is reversed as the applied potential goes back from 1.8 to r1.6 to r1.4 V.

The potential-induced changes in the spin states of catalysts has also been recently observed in the Fe-porphyrin catalysts for the oxygen reduction reaction, where the spin state transition is likely attributed to the changes in the geometry of the Fe–N_*x*_ sites.^47,52^ However, as unveiled from the Fe/Co K-edge EXAFS spectra of CFO from the in situ XAS measurement, there is no significant structural change in the CFO catalyst when a potential bias is applied, indicating that CFO is stable during the OER process. The potential-induced spin transition of CFO during the OER process is believed to be more sensitive at the catalyst–electrolyte interface, not at the species buried in the bulk of catalysts.^15,53^ Therefore, the observed change in the spin states of Fe (Co) 3d ions of the CFO catalyst is mainly attributed to electron transfer from absorbed OH^–^ ions to Fe (Co) 3d states, facilitated by an applying potential. This can be further supported by the decreased intensity of the pre-edge K-edge feature of Fe (Co) of CFO from the in situ XAS measurement, as shown in Figure S11. The decrease in the number of unoccupied states at the Fe (Co) 3d orbitals as the OER occurs signifies electron transfer from absorbed OH^–^ ions to Fe (Co) 3d states, facilitated by the application of a potential bias. The transferred electrons from absorbed OH^–^ ions to Fe (Co) 3d ions of CFO may form intermediates at the catalyst–electrolyte interface during the OER process. As revealed from the above EXAFS spectra, both Fe and Co 3d ions in CFO consist of *T*_d_ A- and O_h_ B-sites spin-aligned in opposite directions. The existence of nonvanishing magnetization both for Fe and Co in CFO due to ferrimagnetic ordering may cause a certain degree of spin alignment with adsorbed OH^–^ ions. Therefore, the overall spin values increase as the number of electrons transferred from absorbed OH^–^ ions to Fe (Co) 3d ions of CFO increases when the potential is increased from 1.4 (before the onset), 1.6 (at the onset), and 1.8 V (after the onset). The spin values of Fe (Co) 3d ions are increased from 2.1 (1.0) to 2.4 (1.2) when the potential is applied from 1.4 to 1.8 V during the OER. A similar variation trend can also be observed when the reverse potential bias is applied from 1.8 to r1.6 to r1.4 V.

When a magnetic field is applied, a more significant spin alignment between Fe/Co ions and adsorbed OH^–^ occurs during the OER process. Consequently, the number of spin-selective electrons transferred from absorbed OH^–^ ions to Fe (Co) 3d ions of CFO further increases in the presence of a magnetic field compared to that without a magnetic field, resulting in increased spin values, as shown in Figure 4c,d. In particular, the enhanced spin values of Co 3d ions are more significant than those of Fe 3d ions during OER electrocatalytic activities under the influence of a magnetic field. The phenomena can be seen in all of the different applied potential biases during the OER process. The result reveals that the spin magnetic effect, where the spin states of OER catalysts can be influenced by an external magnetic field, is more sensitive to Co 3d ions than Fe 3d ions in CFO. The mixed-inverse spinel structure of CFO contains substantial amounts of Fe^3+^/Fe^2+^ ions situated at both the *T*_*d*_ A- and *O*_*h*_ B-sites, with their antiparallel alignment leading to a smaller net magnetic moment due to cancellation.^43^ By contrast, Co ions predominantly occupy the *O*_*h*_ B-sites compared to the *T*_*d*_ A-site. Therefore, the spin-polarization of Co 3d ions of CFO is more responsive than that of Fe 3d ions when an external magnetic field is applied. The result can be further supported by density functional theory (DFT) calculations, which were performed to investigate the spin configurations of different atomic structures in CFO. As revealed from the FT spectra of Fe/Co K-edge EXAFS spectra of CFO in Figure 2c,d, both Fe and Co have the local atomic configurations at the *T*_*d*_ A- and *O*_*h*_ B-sites.

**Figure 5:**
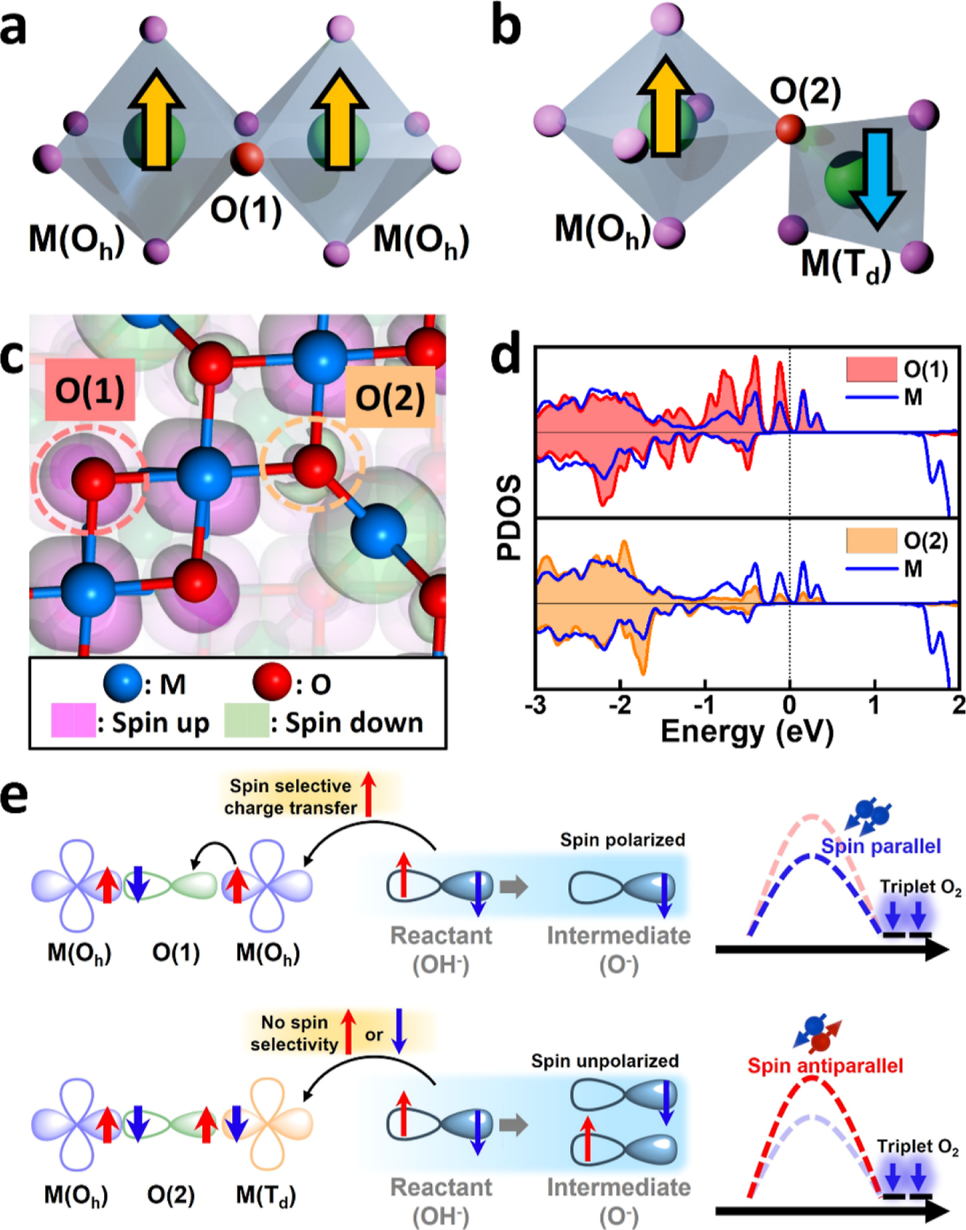
(a) Illustrations of two configurations of M(*O*_*h*_)–O(1)–M(*O*_*h*_) and (b) M(*O*_*h*_)–O(2)–M(*T*_*d*_). (c) Spin density distribution on the CFO (001) surface (pale purple: spin-up; pale green: spin-down). (d) Projected density of states for two different O and transition-metal ions on the CFO (001) surface. (e) Mechanism of the spin-selective charge transfer of adsorbed OH^–^ ions through the M(*O*_*h*_)–O(1)–M(*O*_*h*_) and the M(*O*_*h*_)–O(2)–M(*T*_*d*_) sites in CFO.

Figure 5a,b shows the two representative atomic structures of M(*O*_*h*_)–O(1)–M(*O*_*h*_) and M(*O*_*h*_)–O(2)–M(*T*_*d*_) on the CFO surface, where O(1) and O(2) represent two distinct oxygen atoms adjacent to the metallic ions (either Fe or Co). The metallic ions in the M(*O*_*h*_)–O(1)–M(*O*_*h*_) configuration exhibit parallel spin alignment, whereas those in the M(*O*_*h*_)–O(2)–M(*T*_*d*_) configuration display antiparallel spin alignment owing to the opposite spin alignment of the *O*_*h*_ and *T*_*d*_ sublattices in CFO. Figure 5c exhibits the calculated spin densities of the oxygen atoms on the CFO (001) surface, where O(1) and O(2) represent the two different oxygen atoms neighboring the metallic ions (either Fe or Co) with atomic configurations of M(*O*_*h*_)–O(1)–M(*O*_*h*_) and M(*O*_*h*_)–O(2)–M(*T*_*d*_), respectively. A higher spin density is observed at the O(1) sites in the M(*O*_*h*_)–O(1)–M(*O*_*h*_) configuration compared to that of the O(2) sites in M(*O*_*h*_)–O(2)–M(*T*_*d*_). Figure 5d exhibits the corresponding projected spin density of states, with a stronger overlap between the 3d states of the metal ions (M 3d) and the 2p states of O(1) in the M(*O*_*h*_)–O(1)–M(*O*_*h*_) configuration, compared to the overlap with O(2) in M(*O*_*h*_)–O(2)–M(*T*_*d*_). Calculating the integrated crystal orbital Hamilton population (ICOHP) can further estimate the local bonding strengths.^54^ As shown in Table S3, the ICOHP value associated with O(1)–M(*O*_*h*_) is larger than those observed for O(2)–M(*O*_*h*_) and O(2)–M(*T*_*d*_). This indicates that the bond strength and orbital interaction are stronger in the O(1)–M bond than that in the O(2)–M bonds. This result can also be evident from the shorter bond distances of Fe_oct_–M_oct_/Co_oct_–M_oct_ than those of Fe_tet_–M_oct_/Co_tet_–M_oct_, as shown in Figure 2c,d unveiled by the EXAFS spectra.

According to the QSEI theory,^9^ the reaction kinetics and catalytic activity generally increase when interatomic FM interactions dominate, while it sensibly decreases when AFM interactions prevail. The spin alignment in the M(*O*_*h*_)–O(1)–M(*O*_*h*_) configuration, which resembles the interatomic FM exchange-like interactions as described in the QSEI, can strengthen the Fe (Co) 3d–O 2p hybridization between metal ions and adsorbed OH^–^ ions associated with the Fermi holes.^55^ As a result, it will facilitate spin-selected charge transport with smaller electron–electron repulsion and optimize the kinetics of the spin-charge transfer at catalyst–electrolyte interfaces. By contrast, the M(*O*_*h*_)–O(1)–M(*T*_*d*_) configuration with antiparallel spin alignment, resembling the AFM exchange-like interactions, exhibits decreased reaction kinetics with less efficient spin-selective charge transfer.^56^Figure 5e illustrates the mechanism of spin-selective charge transfer for adsorbed OH^–^ ions through the M(*O*_*h*_)–O(1)–M(*O*_*h*_) and M(*O*_*h*_)–O(2)–M(*T*_*d*_) structures in CFO. The M(*O*_*h*_)–O(1)–M(*O*_*h*_) pathway is preferred for transferring spin-polarized electrons from the OH^–^ ions, resulting in intermediates (O^–^) with aligned spins and ultimately forming a triplet oxygen state. Conversely, the M(*O*_*h*_)–O(2)–M(*T*_*d*_) site lacks spin selectivity, allowing the formation of both spin-up and spin-down intermediates without spin-polarization. Because the spin-polarized intermediates have a lower energy barrier for the OER compared to the spin-unpolarized counterparts, the M(*O*_*h*_)–O(1)–M(*O*_*h*_) configuration exhibits enhanced reaction kinetics with more efficient spin-selective charge transfer and the OER catalytic activity compared to the M(*O*_*h*_)–O(2)–M(*T*_*d*_) configuration. The EXAFS spectra shown in Figure 2c,d indicate that Fe and Co ions in the CFO consist of both M(*O*_*h*_)–O(1)–M(*O*_*h*_) and M(*O*_*h*_)–O(2)–M(*T*_*d*_) atomic configurations, where a higher proportion of the M(*O*_*h*_)–O(1)–M(*O*_*h*_) configuration is present in both Fe and Co. When a potential bias is applied to the CFO catalyst in the absence of a magnetic field, spin-selected charge transfer for adsorbed OH^–^ reactants primarily occurs through the M(*O*_*h*_)–O(1)–M(*O*_*h*_) sites for both Fe and Co 3d ions. As a result, increased average spin values for both Fe and Co are observed in the in situ XES spectra as the potential is increased from 1.4 to 1.6 and 1.8 V during the OER, as shown in Figure 4c,d. By contrast, when an external magnetic field is applied to the CFO catalyst during the OER, the increase in the average spin values is greater for Co than that for Fe. This discrepancy arises from the more efficient spin-selective charge transfer occurring in Co during OER under the influence of the external magnetic field. Because Co ions have a higher portion of M(*O*_*h*_)–O(1)–M(*O*_*h*_) atomic configuration with a more ordered spin-aligned structure compared to the Fe counterpart, the spin-polarization of Co 3d ions of the CFO is more responsive than that of Fe 3d ions when an external magnetic field is applied. The previous hypothesis suggests that magnetic field-enhanced OER of CFO only occurs at the Co sites, where Co in *O*_*h*_ positions contributes to the effective magnetic moment.^15^ In this study, we employed in situ XES featuring the distinct *K*_β′_ emission fingerprints to uncover the individual influence of spin states of Fe and Co 3d ions in CFO on the catalytic activities of the OER under the effects of an electrical potential bias and an external magnetic field. Both Fe and Co play important roles in enhancing spin-selective charge transfer during the OER when subjected to an external magnetic field. This enhanced performance is mainly attributed to the spin magnetic effect of Fe and Co 3d ions, which arises from the ferrimagnetic ordering and the spin configurations of Fe and Co ions in CFO. To the best of our knowledge, this study provides the first experimental evidence revealing the correlation between the spin magnetic effect and the atomic/electronic structures of an OER catalyst with an external magnetic field under in situ conditions.

## 3 Conclusions

In conclusion, we utilized in situ XES and XAS techniques to elucidate the relationship among atomic/electronic structures, spin states, and the OER catalytic activities of the CFO catalyst under an external magnetic field. The intrinsic nonvanishing magnetization of both Fe and Co, stemming from ferrimagnetic ordering, facilitates spin-selective electron transfer from adsorbed OH^–^ reactants. This effect is further enhanced when an external magnetic field is applied. The spin polarization of Co 3d ions in CFO is more responsive to an external magnetic field than that of Fe 3d ions, which can be attributed to a higher portion of the M(*O*_*h*_)–O(1)–M(*O*_*h*_) atomic structure with a spin-aligned configuration in Co ions compared to their Fe counterparts. This work paves the way for in situ/operando studies of FM and ferrimagnetic catalysts under external magnetic fields, providing a new insight into the influence of spin magnetic effects on OER catalysts.

## Supporting Information Available

Experimental methods, computational methods, additional characterization data (XRD, Raman, and EDS), photographs of the magnetic field-enhanced electrocatalysis setup and liquid cell for in situ XES measurement, in situ XAS spectrum (FT-EXAFS), and in situ XES spectrum (reverse potential) (PDF)
