# Supplementary material for: In Situ Identification of Spin Magnetic Effect on Oxygen Evolution Reaction Unveiled by X-ray Emission Spectroscopy
Source: J Am Chem Soc. 2025 Apr 12;147(16):13286–95. doi: 10.1021/jacs.4c18149 (PMC12022985; doi:10.1021/jacs.4c18149)
Supplement: Supplementary file 1 — ja4c18149_si_001.pdf [file ja4c18149_si_001.pdf]

Supporting Information

In situ Identification of Spin Magnetic Effect on Oxygen Evolution Reaction Unveiled by X-ray Emission Spectroscopy

Chih-Ying Huang,[a,b] Hsin-An Chen,[c] Wei-Xuan Lin,[d] Kuan-Hung Chen,[d] Yu-Chang Lin,[e] Tai-Sing Wu,[e] Chia-Che Chang,[e] Chih-Wen Pao,[e] Wei-Tsung Chuang,[e] Jyh-Chyuan Jan,[e] Yu-Cheng Shao,[e] Nozomu Hiraoka,[e] Jau-Wern Chiou,[f] Pai-Chia Kuo,[g] Jessie Shiue,[g] Deepak Vishnu S. K,[b,h] Raman Sankar,[i] Zih-Wei Cyue,[j] Way-Faung Pong,[d,*] and Chun-Wei Chen[a,j,k,*]

a International Graduate Program of Molecular Science and Technology (NTU-MST), National Taiwan University, Taipei 106319, Taiwan

b Molecular Science and Technology Program Taiwan International Graduate Program (TIGP), Academia Sinica, Taipei 115201, Taiwan

c Institute of Materials Science and Engineering, National Taipei University of Technology, Taipei 10608, Taiwan

d Department of Physics, Tamkang University, New Taipei City 251301, Taiwan

e National Synchrotron Radiation Research Center, Hsinchu 300, Taiwan

f Department of Applied Physics, National University of Kaohsiung, Kaohsiung 811, Taiwan

g Institute of Atomic and Molecular Sciences, Academia Sinica, Taipei 106319, Taiwan

h Department of Chemistry, National Tsing Hua University, Hsinchu 300, Taiwan

i Institute of Physics, Academia Sinica, Taipei 115201, Taiwan

j Department of Materials Science and Engineering, National Taiwan University, Taipei 106319, Taiwan

k Center for Condensed Matter Sciences and Center of Atomic Initiative for New Materials (AI-MAT), National Taiwan University (NTU), Taipei 106319, Taiwan

Materials synthesis

The $CoFe_2O_4$ (CFO) nanocrystals were synthesized using a hydrothermal method. Initially, 1.08 g of $FeCl{\cdot}6H_2O$ (4 mmol), 0.48 g of $CoCl_2{\cdot}6H_2O$ (2 mmol), and 0.8 g of NaOH (20 mmol) were dissolved in 7 mL of deionized water at room temperature. Subsequently, 14 mL of ethylenediamine was slowly added. The mixture was then transferred into a Teflon-lined autoclave and heated at 110 °C for 15 hours. After cooling to room temperature, the solution was filtered and washed with deionized water at least 5 times. Finally, the CFO nanocrystals were obtained through centrifugation.

Materials characterization

The powder X-ray diffraction patterns were measured by using Bruker D8 with Cu K_α radiation (λ = 1.5418 Å). The Raman spectra were performed on a confocal Raman microscope with a 532 nm laser and a 50× microscope objective with a numerical aperture of 0.55. The atomic images were taken by Cs-corrected scanning transmission electron microscopy (JEOL JEM-ARM300F2) at an accelerated voltage of 300 kV. The composition of CFO was determined by energy-dispersive X-ray spectroscopy experiments carried out in the STEM. The magnetic measurements were conducted with a Quantum Design VSM-SQUID magnetometer.

Electrochemical characterization

The OER tests were operated in a three-electrode cell with a working electrode of carbon fiber paper (10 × 20 × 0.5 mm; effective electrode area: 1.0 cm^2), a counter electrode of platinum wire, and an Ag/AgCl reference electrode (filled with 3 M KCl solution). The catalyst electrode was fabricated using the drop-casting method. The CFO and carbon black were dispersed in isopropanol/water (v/v =1:4) solvent, followed by adding Na^+-exchanged Nafion as the binder. The mixtures were ultrasonicated for 30 min to reach a homogeneous solution. The concentration of CFO in solution is 5 mg mL^{-1}, and carbon black is 1 mg mL^{-1}. Finally, the as-prepared solution (500 µL) was dropped onto carbon fiber paper electrodes and dried at room temperature. Linear sweep voltammetry (LSV) curves were performed in O_2-saturated 1 M $KOH_{(aq)}$ by using Autolab PGSTAT204. LSV measurements for OER activity were recorded with a scan rate of 5 mV s^{-1} in a potential range of 1.4 to 1.8 V vs. RHE. Electrochemical impedance spectroscopy (EIS) was performed at frequencies between 10 kHz and 0.01 Hz with an overpotential of 400 mV (about 1.6 V vs. RHE). All potentials were converted to the RHE scale according to the following equation: E(RHE) = E(Ag/AgCl) + 0.207 V + 0.059 V × pH with 90% iR correction. The

uncompensated resistances (R_u) were determined by EIS measurement, as shown in **Tables S1** and **S2**. **Figures S15** and **S16** show the LSV curves of CFO with and without iR correction in the typical reactor and in situ XES cell, respectively. The pH value (14 for 1 M KOH) was determined using a pH meter. We performed LSV of CFO under the magnets (0.4 T) to examine the magnetically enhanced OER. The experimental procedures are provided below: (1) Scan LSV in a potential range of 1.4 to 1.8 V vs. RHE continuously until stable; (2) Move the magnets close to the working electrode and wait 10 min. The setup is shown in **Figure S12**; (3) Scan LSV under the magnetic field until stable. The result is shown in **Figure S13**. In addition, when considering magnetic field-assisted electrocatalysis, several effects, including the magnetothermal effect, mass transfer, and spin selectivity effect, should be considered.[1, 2] Firstly, the magnetothermal effect can be neglected because this work used a permanent magnet of 0.4T for the external magnetic field. All experiments were operating at a constant temperature and no heating process. Secondly, mass transfer capacity is important for diffusing reactants and products. The accumulation of bubbles can block the interface between electrolytes and catalysts, which means boosting the leaving of bubbles on the electrode surface can effectively enhance the performance.[3] Some reports show that the magnetohydrodynamic effect can improve the release of bubbles under high overpotential and high current conditions.[4, 5] Due to the current density of CFO being relatively small in our experiment, oxygen bubble generation is not intense and leaves smoothly when a magnetic field is applied.

Synchrotron radiation-based spectroscopic measurements – XAS and XES

The in situ Fe (Co) *K*-edge XANES and EXAFS spectra of CFO were carried out at the Taiwan Photon Source (TPS) 44A beamline in transmission mode at the National Synchrotron Radiation Research Center (NSRRC), Taiwan. The energy calibration for Fe (Co) *K*-edge XANES and EXAFS data was performed by measuring the Fe (Co) foil reference during the spectroscopic measurements. All FT-EXAFS spectra were phase-corrected and obtained from k^3-weighted $\chi(k)$ data. In situ Fe (Co) K_β XES of CFO were measured at the Taiwan inelastic X-ray scattering beamline BL12XU at SPring-8, Japan. The undulator beam was made monochromatic with two Si(111) crystals and focused on the sample position with two Kilpatrick-Baez (KB) focusing mirrors. The Fe (Co) K_β XES was collected at 90° from the incident X-ray and analyzed with a spectrometer (Johann-type) equipped with a spherically bent Ge(444) crystal and a solid-state detector arranged on a horizontal plane in a Rowland-circle geometry.

For the in situ Fe (Co) *K*-edge XANES/EXAFS and K_β XES measurements, the working

electrodes were prepared separately with corresponding redox potential, mounted on the sample holder with a magnetic field of 0.4 T. **Figure S12** shows the relative position of liquid cell, magnets, and X-ray.

We evaluate the spin values of Co and Fe ions in CFO by calculating the $K_{\beta'}$ area. Firstly, the $K_{\beta'}$ area for Fe (Co) was obtained by fitting a Gaussian peak in the energy range of 7034 to 7049 eV (7629 to 7641 eV) from the background-subtracted emission spectra, orange line in **Figure S14 a**. Then, divide the $K_{\beta'}$ area by the overall K_β area to obtain the relative $K_{\beta'}$ area. Based on previous literature, we established a linear regression using reference compounds with known spin values compared to the emission spectra we measured. The reference compounds include FeO, Fe_3O_4, Fe_2O_3, $LiCoO_2$, Co_3O_4, and CoO. By substituting the relative $K_{\beta'}$ area of CFO into this linear regression, we can determine the spin value for CFO. This is the typical way of XES data analysis. More details of data treatment can be found in previous studies.[6, 7]

For instance, the spin of CFO under 1.4 V was calculated below:

The relative area: $R.A.\ (\%) = \frac{0.3319\ (Area\ of\ K_{\beta'})}{8.9693\ (Area\ of\ overall\ K_{\beta})} \times 100\% = 3.7\%$

The linear function of Co: $Spin = 0.25R.A. + 0.078$

The Co spin value of CFO under 1.4 V: $Spin = 0.25 \times 3.7 + 0.078 = \mathbf{1.0}$

This article's "spin value" is "nominal spin, S," referring to the work that P. Glatzel's group reported.[8] Nominal spin values are derived from an ionic model, considering the formal valence and the crystal field splitting. For the monovalent samples, FeO (Fe^{2+}, d^6), S = 2; Fe_2O_3 (Fe^{3+}, d^5), S = 2.5; $LiCoO_2$ (Co^{3+}, d^6), S = 0; CoO (Co^{2+}, d^7), S = 1.5.

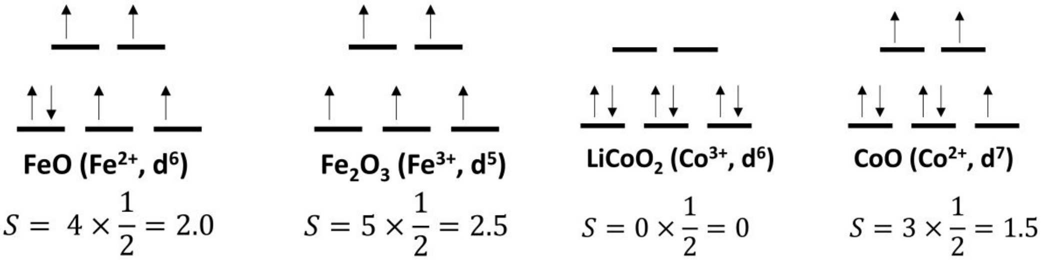

For the mixed-valence samples, Fe_3O_4 and Co_3O_4, the non-integer average valence is based on the site occupation of formal integer valences normalized by the number of Fe and Co atoms in the chemical formula. The nominal spin is then calculated by assigning the corresponding S value to each integer valence.

For example, Fe_3O_4 has two sites occupied by Fe^{2+} and Fe^{3+} ions. Therefore, the spin value of Fe_3O_4 can be calculated by $\frac{1}{3}\left[1 \times Fe^{3+} + 2\left(\frac{1}{2} \times Fe^{3+} + \frac{1}{2} \times Fe^{2+}\right)\right] = \frac{1}{3}\left[1 \times 2.5 + 2\left(\frac{1}{2} \times 2.5 + \frac{1}{2} \times 2.0\right)\right] = 2.3$, $\boldsymbol{S_{Fe_3O_4} = 2.3}$

The spin value of Co_3O_4 can be calculated by $\frac{1}{3}\left[1 \times Co^{3+} + 2\left(\frac{1}{2} \times Co^{3+} + \frac{1}{2} \times Co^{2+}\right)\right] = \frac{1}{3}$ $\left[1 \times 0 + 2\left(\frac{1}{2} \times 0 + \frac{1}{2} \times 1.5\right)\right] = 0.5$, $\boldsymbol{S_{Co_3O_4} = 0.5}$

Computational method

In this research, *ab initio* calculations of the cleaved (001) surface of inverse spinel CFO were performed using the Vienna *ab initio* Simulation Package (VASP) based on density functional theory (DFT).[9-11] The generalized gradient approximation (GGA) was used with the Perdew-Burke-Ernzerhof (PBE)[12, 13] exchange-correlation functional and the projector augmented wave (PAW)[14, 15] pseudopotentials. The cutoff energy was set to be 400 eV, and the self-consistent field convergence criterion for energy was set to be 10^{-5} eV. The electronic structure was fully optimized until the forces of all ions were smaller than 10^{-2} eV $Å^{-1}$. To properly describe the strong on-site Coulomb interactions presented in the *d*-orbitals of transition metals oxide, the DFT+U method was employed in all calculations with Hubbard U values of 2.8 eV for Co and 3.1 eV for Fe, respectively.[16, 17] 4x4x1 Γ-centered *k*-point mesh was used in the geometry optimization, whereas 6x6x1 Γ-centered k-point meshes were used in the spin density distribution and the density of states calculation. No band gap correction was applied.

We have calculated the values of the Integrated Crystal Orbital Hamilton Population (ICOHP) according to the Reviewer's suggestion. In the analysis of bond strength between the O and M sites with respect to their predominant spin states, we calculated the Integrated Crystal Orbital Hamilton Population (-ICOHP) values, as shown in **Table S3**. The ICOHP value associated with the spin-up states in the O(1)-M is greater than that observed in O(2)-M, indicating that the bond strength, as well as the orbital interaction, is stronger in O(1)-M in comparing to O(2)-M. The results from the ICOHP analyses are consistent with the projected density of states, providing more comprehensive and robust insight from the theoretical viewpoint.

Figures

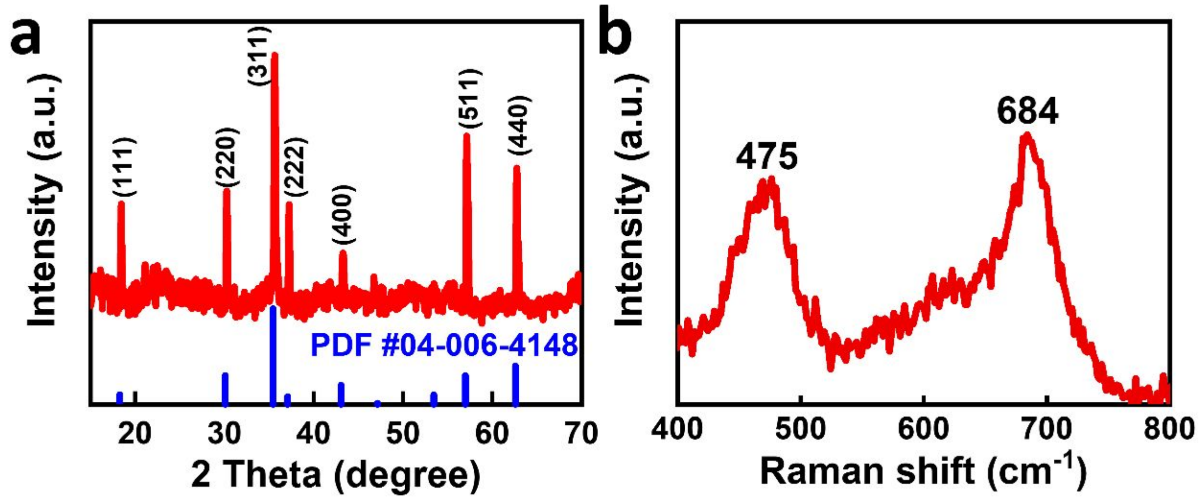

Figure S1. (a) XRD pattern of CFO with inverse spinel reference. (b) Raman spectra of CFO.

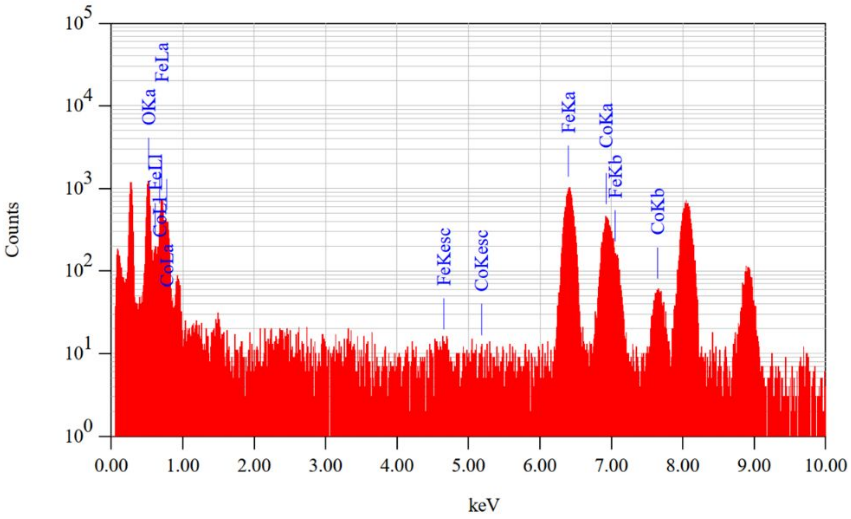

Figure S2. (a) EDS analysis of CFO.

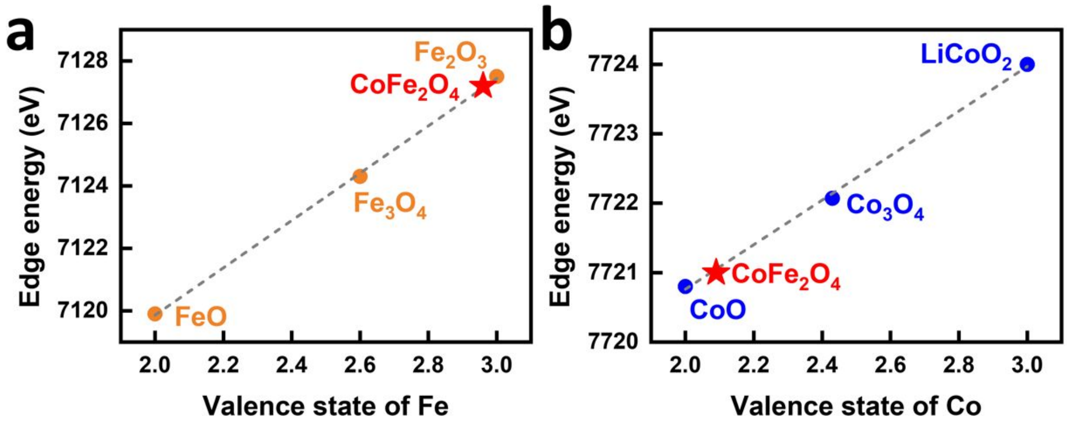

Figure S3. (a) Fe and (b) Co calibration curves valence state of CFO by *K*-edge energy with their corresponding reference samples.

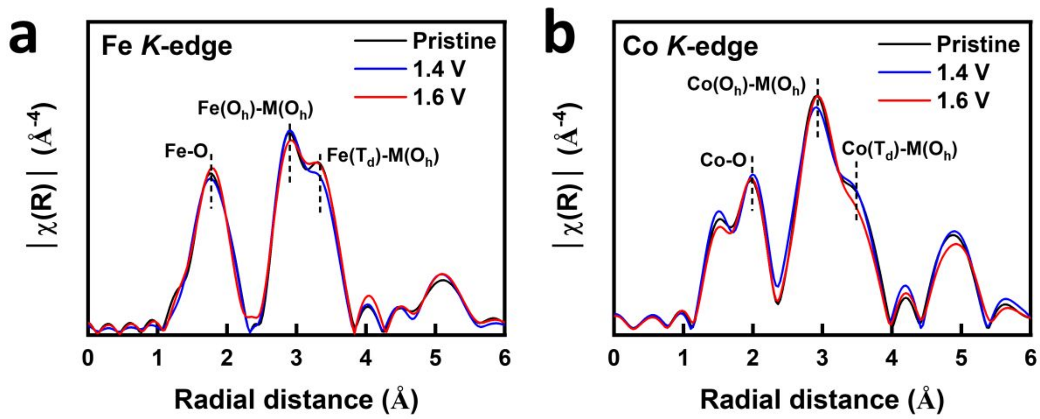

Figure S4. (a) Phase-corrected FT-EXAFS of Fe and (b) Co *K*-edge of pristine CFO and under an applied bias obtained from k^3-weighted $\chi(k)$ data.

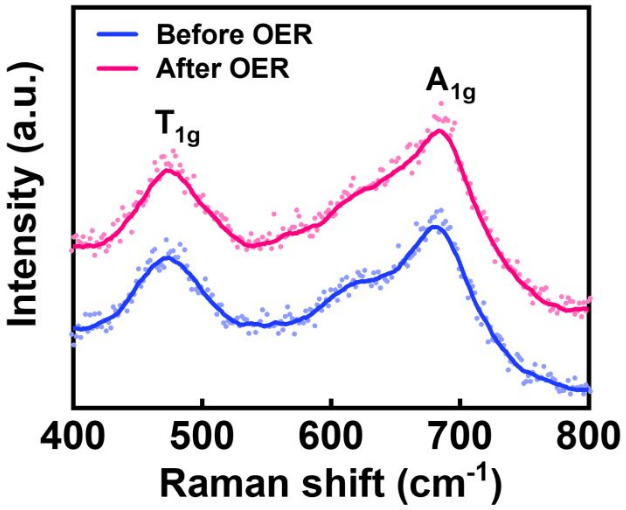

Figure S5. Raman spectra of CFO before and after OER reaction.

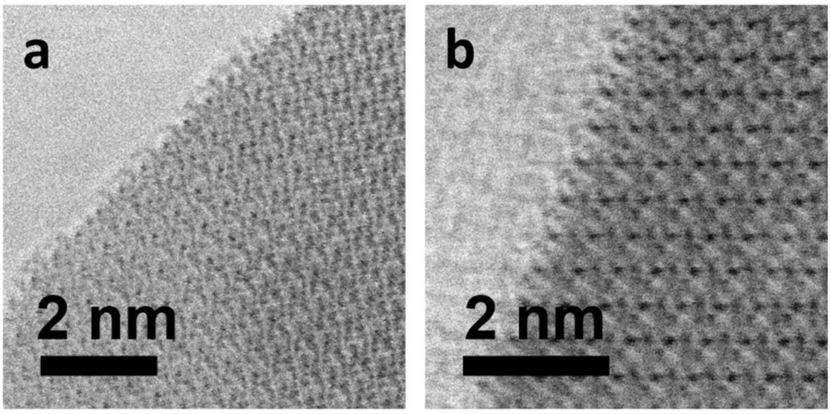

Figure S6. STEM images of CFO (a) before and (b) after OER reaction.

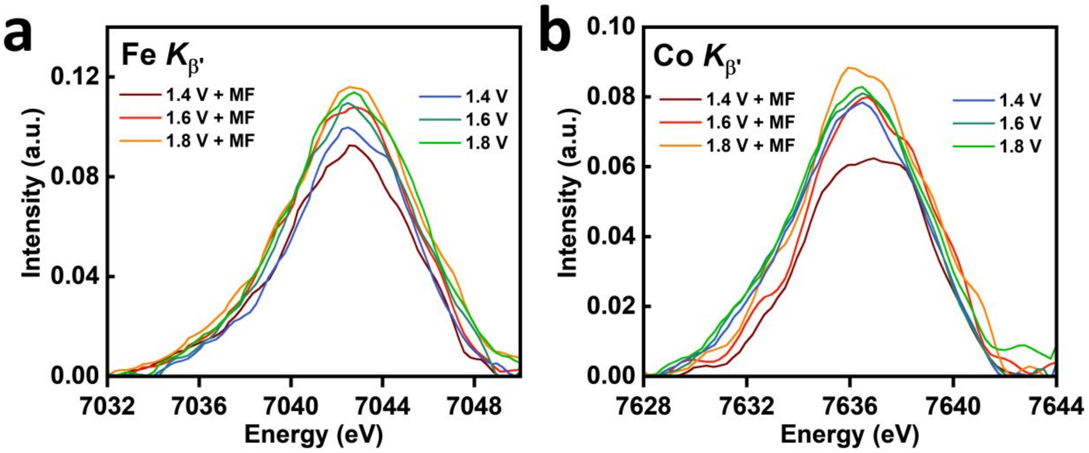

Figure S7. (a) Fe and (b) Co $K_{\beta'}$ XES under different potentials with and without an applied magnetic field.

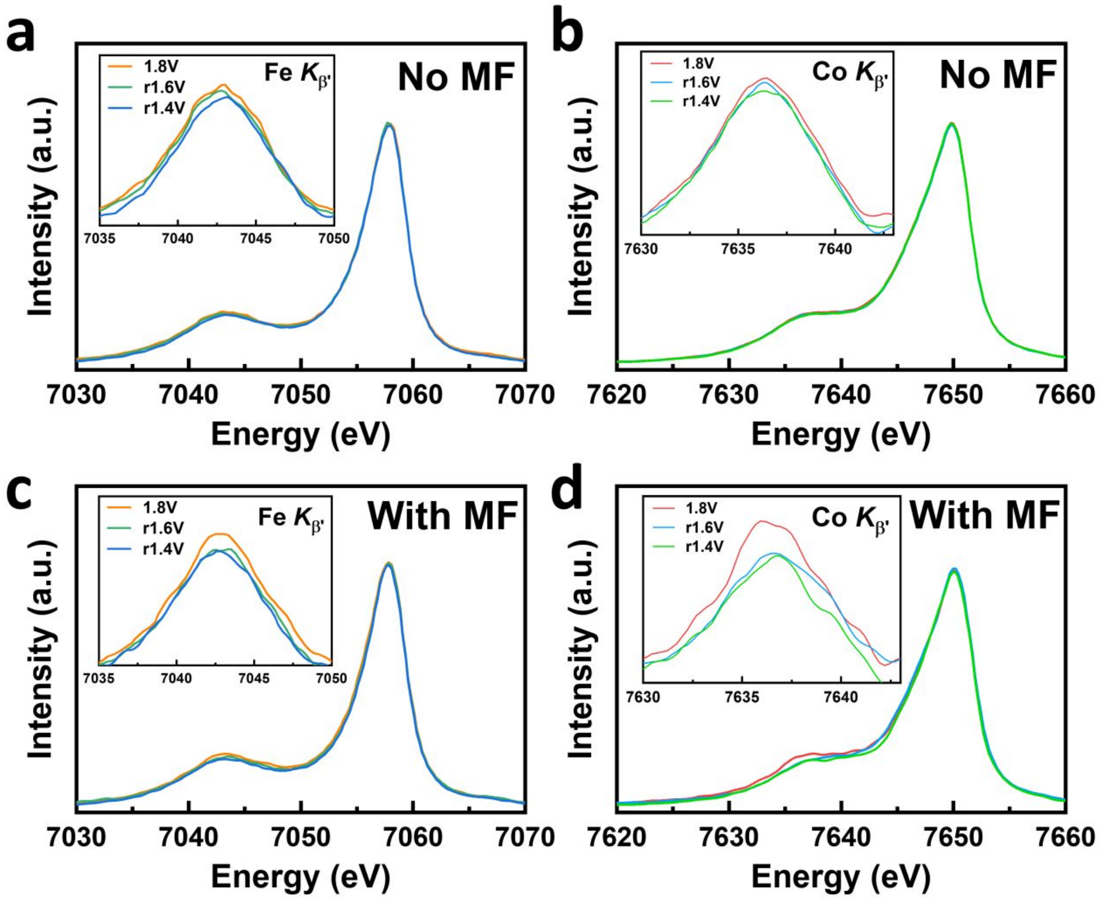

Figure S8. (a) Fe and (b) Co K_{β} XES of CFO under reverse potential applied no magnetic field (MF). (c) Fe and (d) Co K_{β} XES of CFO under reverse potentials with a magnetic field applied. Insets present the corresponding $K_{\beta'}$ emission spectra after background subtraction.

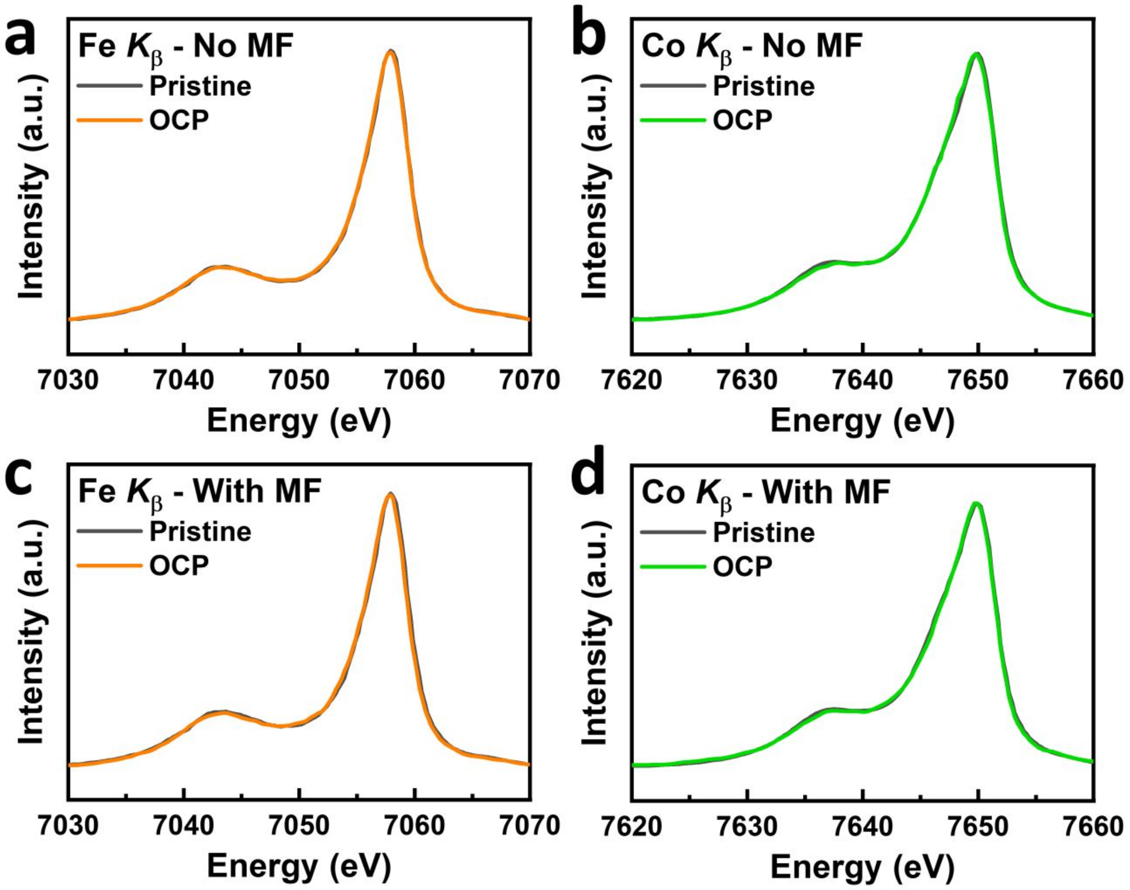

Figure S9. (a) Fe and (b) Co K_β XES of pristine CFO and at OCP. (c) Fe and (d) Co K_β XES of pristine CFO and at OCP with a magnetic field applied.

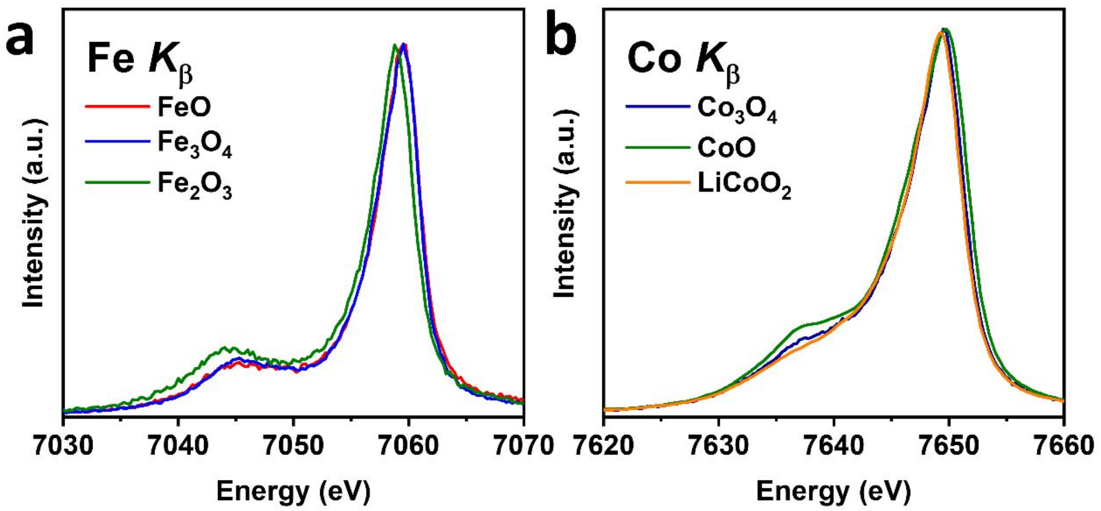

Figure S10. (a) Fe and (b) Co K_β XES of reference samples.

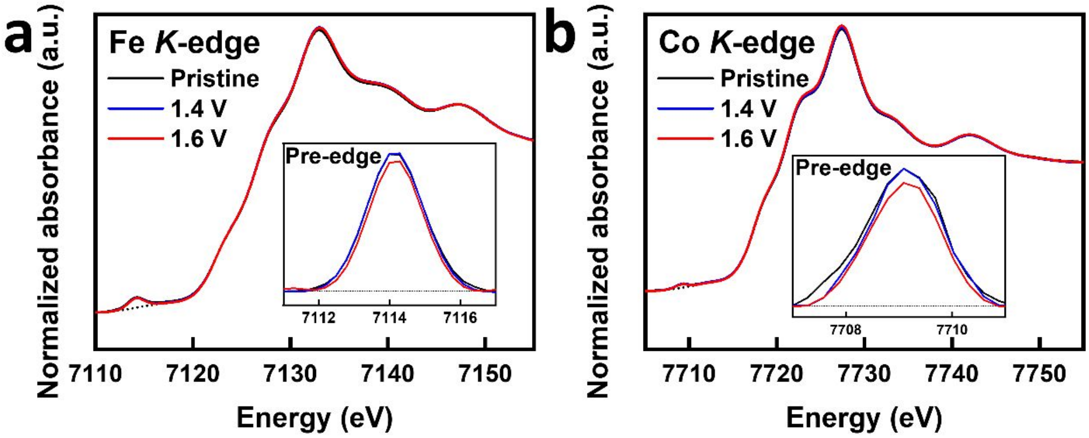

Figure S11. (a) Fe and (b) Co *K*-edge XANES of pristine CFO and under different applied potentials. Insets present the pre-edge feature after the background subtraction (dashed line).

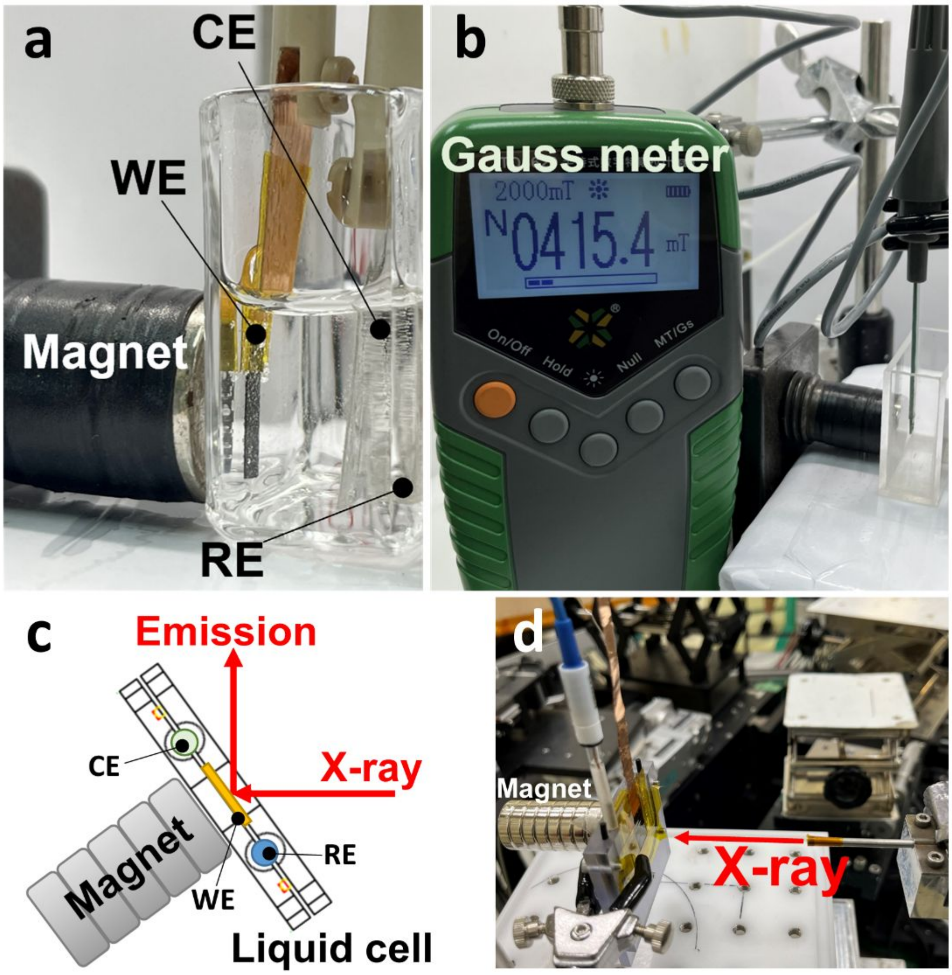

Figure S12. (a) and (b) Photos of the magnetic field-enhanced electrocatalysis setup. The strength of the magnetic field was measured by a Gauss meter. (c) Illustration of the liquid cell and magnets for XES measurement. (d) Photo of the liquid cell on the XES measurement stage.

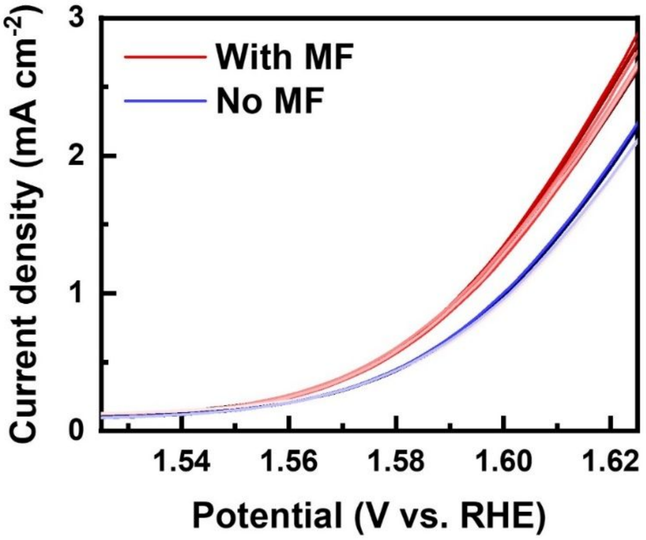

Figure S13. LSV curves of CFO with and without magnetic field.

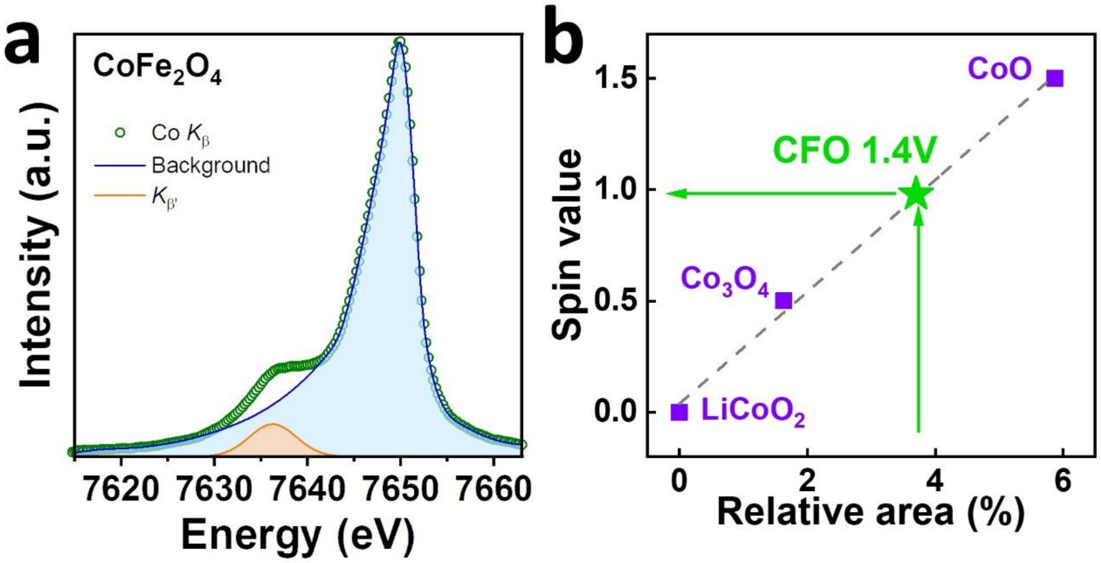

Figure S14. (a) XES of Co in CFO under 1.4 V. (b) Linear regression of Co.

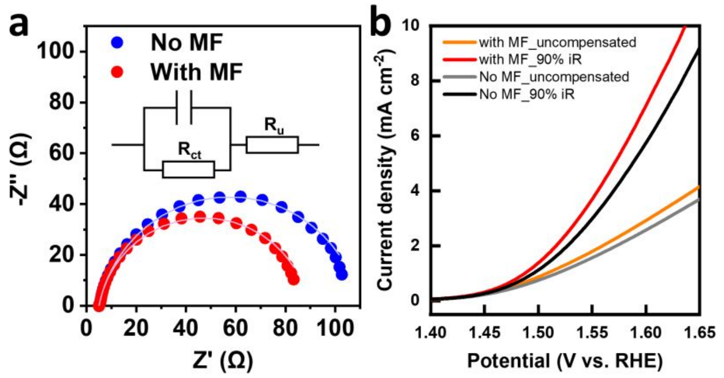

Figure S15. (a) Nyquist plots of CFO with and without a magnetic field applied, measured in the typical reactor. (b) The LSV curves with and without iR correction.

Table S1. The uncompensated (R_u) and charge transfer resistances (R_{ct}) of the CFO catalyst with and without MF were measured in the typical reactor.

**Condition**	R_u	R_{ct}
No MF	5.02	105.2
With MF	5.14	84.1

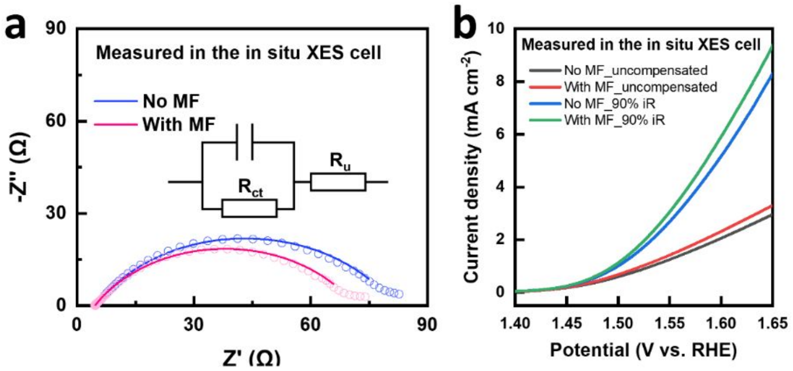

Figure S16. (a) Nyquist plots of CFO with and without magnetic field applied, measured in the in situ XES cell. (b) The LSV curves were measured in the in situ XES cell with and without iR correction.

Table S2. The uncompensated (R_u) and charge transfer resistances (R_{ct}) of the CFO catalyst with and without MF were measured in the in situ XES cell.

**Condition**	R_u	R_{ct}
No MF	4.61	77.1
With MF	4.54	66.9

Table S3. The negative Integrated Crystal Orbital Hamilton Population (-ICOHP) of spin-up M_{3d}-O_{2p} with different O(1) and O(2).

Type	-ICOHP
O(1)-M	0.2828
O(2)-M	0.2731

References

(1) Luo, S.; Elouarzaki, K.; Xu, Z. J. Electrochemistry in Magnetic Fields. *Angew. Chem. Int. Ed.* **2022**, *61* (27), e202203564. DOI: 10.1002/anie.202203564

(2) Ma, S.; Fu, Q.; Han, J.; Yao, T.; Wang, X.; Zhang, Z.; Xu, P.; Song, B. Magnetic Field-Assisted Water Splitting: Mechanism, Optimization Strategies, and Future Perspectives. *Adv. Funct. Mater.* **2024**, *34* (26), 2316544. DOI: 10.1002/adfm.202316544

(3) Sun, Z.; Wang, Y.; Zhang, L.; Wu, H.; Jin, Y.; Li, Y.; Shi, Y.; Zhu, T.; Mao, H.; Liu, J.; et al. Simultaneously Realizing Rapid Electron Transfer and Mass Transport in Jellyfish-Like Mott–Schottky Nanoreactors for Oxygen Reduction Reaction. *Adv. Funct. Mater.* **2020**, *30* (15), 1910482. DOI: https://doi.org/10.1002/adfm.201910482

(4) Matsushima, H.; Iida, T.; Fukunaka, Y. Gas bubble evolution on transparent electrode during water electrolysis in a magnetic field. *Electrochim. Acta* **2013**, *100*, 261-264. DOI: https://doi.org/10.1016/j.electacta.2012.05.082

(5) Monzon, L. M. A.; Nair, V.; Reilly, B.; Coey, J. M. D. Magnetically-Induced Flow during Electropolishing. *J. Electrochem. Soc.* **2018**, *165* (13), E679. DOI: 10.1149/2.0581813jes

(6) Vankó, G.; Neisius, T.; Molnár, G.; Renz, F.; Kárpáti, S.; Shukla, A.; de Groot, F. M. F. Probing the 3d Spin Momentum with X-ray Emission Spectroscopy: The Case of Molecular-Spin Transitions. *J. Phys. Chem. B.* **2006**, *110* (24), 11647-11653. DOI: 10.1021/jp0615961

(7) Vankó, G.; de Groot, F. M. F. Comment on ``Spin crossover in (Mg,Fe)O: A Mössbauer effect study with an alternative interpretation of x-ray emission spectroscopy data''. *Phys. Rev. B.* **2007**, *75* (17), 177101. DOI: 10.1103/PhysRevB.75.177101

(8) Lafuerza, S.; Carlantuono, A.; Retegan, M.; Glatzel, P. Chemical Sensitivity of K_{β} and K_{α} X-ray Emission from a Systematic Investigation of Iron Compounds. *Inorg. Chem.* **2020**, *59* (17), 12518-12535. DOI: 10.1021/acs.inorgchem.0c01620

(9) Kresse, G.; Hafner, J. Ab initio molecular dynamics for liquid metals. *Phys. Rev. B.* **1993**, *47* (1), 558-561. DOI: 10.1103/PhysRevB.47.558

(10) Kresse, G.; Hafner, J. Ab initio molecular dynamics for open-shell transition metals. *Phys. Rev. B.* **1993**, *48* (17), 13115-13118. DOI: 10.1103/PhysRevB.48.13115

(11) Kresse, G.; Hafner, J. Ab initio molecular-dynamics simulation of the liquid-metal--amorphous-semiconductor transition in germanium. *Phys. Rev. B.* **1994**, *49* (20), 14251-14269. DOI: 10.1103/PhysRevB.49.14251

(12) Perdew, J. P.; Chevary, J. A.; Vosko, S. H.; Jackson, K. A.; Pederson, M. R.; Singh, D. J.;

Fiolhais, C. Atoms, molecules, solids, and surfaces: Applications of the generalized gradient approximation for exchange and correlation. *Phys. Rev. B.* **1992**, *46* (11), 6671-6687. DOI: 10.1103/PhysRevB.46.6671
(13) Perdew, J. P.; Wang, Y. Accurate and simple analytic representation of the electron-gas correlation energy. *Phys. Rev. B.* **1992**, *45* (23), 13244-13249. DOI: 10.1103/PhysRevB.45.13244
(14) Blöchl, P. E. Projector augmented-wave method. *Phys. Rev. B.* **1994**, *50* (24), 17953-17979. DOI: 10.1103/PhysRevB.50.17953
(15) Kresse, G.; Joubert, D. From ultrasoft pseudopotentials to the projector augmented-wave method. *Phys. Rev. B.* **1999**, *59* (3), 1758-1775. DOI: 10.1103/PhysRevB.59.1758
(16) Anisimov, V. I.; Zaanen, J.; Andersen, O. K. Band theory and Mott insulators: Hubbard U instead of Stoner I. *Phys. Rev. B.* **1991**, *44* (3), 943-954. DOI: 10.1103/PhysRevB.44.943
(17) Dudarev, S. L.; Botton, G. A.; Savrasov, S. Y.; Humphreys, C. J.; Sutton, A. P. Electron-energy-loss spectra and the structural stability of nickel oxide: An LSDA+U study. *Phys. Rev. B.* **1998**, *57* (3), 1505-1509. DOI: 10.1103/PhysRevB.57.1505
